# Exploring Rare and Atypical Forms of Psoriasis: A Narrative Review

**DOI:** 10.3390/jcm15145537

**Published:** 2026-07-15

**Authors:** Federico Bardazzi, Giorgio De Benedetto, Giacomo Clarizio, Lidia Sacchelli, Alessio Natale, Marco Adriano Chessa, Iria Neri, Michelangelo La Placa, Riccardo Balestri

**Affiliations:** 1 Dermatology Unit, IRCCS Azienda Ospedaliero, Universitaria di Bologna, 40138 Bologna, Italy; federico.bardazzi@aosp.bo.it (F.B.); giorgio.debenedetto@studio.unibo.it (G.D.B.); lidia.sacchelli@gmail.com (L.S.); alessio.natale@studio.unibo.it (A.N.); marcoadriano.chessa2@unibo.it (M.A.C.); iria.neri@aosp.bo.it (I.N.); michelangelo.laplaca@unibo.it (M.L.P.); 2 Department of Medical and Surgical Sciences, Alma Mater Studiorum University of Bologna, 40138 Bologna, Italy; 3 Division of Dermatology, Psoriasis Outpatient Service, Azienda Sanitaria Universitaria Integrata del Trentino (ASUIT)—Trento Hospital, 38122 Trento, Italy

**Keywords:** psoriasis, atypical psoriasis, rare psoriasis variants

## Abstract

Psoriasis is a chronic immune-mediated inflammatory dermatosis characterized by marked clinical heterogeneity. Although plaque psoriasis represents the most common phenotype, several uncommon and atypical variants may pose considerable diagnostic challenges because of their unusual morphology, distribution, and clinicopathological presentation. Early recognition of these rare forms is essential to avoid misdiagnosis and inappropriate management. This narrative review summarizes the current evidence regarding selected atypical variants of psoriasis, including psoriasis gyrata, linear psoriasis, follicular psoriasis, psoriatic onycho-pachydermo-periostitis, psoriatic neurodermatitis, interdigital psoriasis, parakeratosis pustulosa, psoriasis of the lips, psoriasiform acral dermatitis, psoriasis dermatitis, and extreme hyperkeratotic forms. For each entity, we discuss epidemiology, clinical presentation, dermoscopic and histopathological findings, differential diagnosis, pathophysiological hypotheses, and therapeutic considerations. Particular attention is given to distinguishing these entities from inflammatory, infectious, and neoplastic mimickers. The integration of clinical evaluation with dermoscopy and histopathological examination remains crucial in challenging cases. Awareness of these uncommon variants may improve diagnostic accuracy and facilitate timely therapeutic intervention.

## 1. Introduction

Psoriasis is a chronic inflammatory skin disease affecting approximately 2–3% of the global population and is currently recognized as a complex immune-mediated disorder involving genetic susceptibility, environmental triggers, and dysregulated innate and adaptive immune responses [1]. While chronic plaque psoriasis is the most prevalent phenotype, psoriasis encompasses a broad clinical spectrum including guttate, erythrodermic, inverse, pustular, and palmoplantar variants. The considerable heterogeneity in morphology, anatomical distribution, clinical course, and therapeutic response among these phenotypes supports the concept of psoriasis as a spectrum of related diseases rather than a single nosological entity [2]. In most cases, the diagnosis of psoriasis is established clinically; however, rare and atypical forms may closely mimic infectious, inflammatory, or neoplastic dermatoses. Histology can become crucial in the diagnosis of these more complex forms of psoriasis, which can present a clinical challenge even to trained dermatologists.

This narrative review aims to provide a comprehensive overview of rare and atypical variants of psoriasis, focusing on their clinical manifestations, differential diagnosis, dermoscopic and histopathological characteristics, pathogenic mechanisms, and available therapeutic strategies.

## 2. Methods

A narrative review of the literature was performed using PubMed/MEDLINE and Scopus databases, with no time restrictions. Articles published in English and relevant historical reports were screened using combinations of the following keywords: “rare psoriasis”, “atypical psoriasis”, “linear psoriasis”, “follicular psoriasis”, “psoriasis gyrata”, “psoriatic neurodermatitis”, “interdigital psoriasis”, “parakeratosis pustulosa”, and “psoriatic onycho-pachydermo-periostitis”. Case reports, case series, observational studies, and review articles were considered. Additional references were identified from the bibliographies of selected articles. After removing duplicates, 39 articles were included in the final synthesis. This review was not conducted as a systematic review.

Bibliometrics was used to conduct frequency analysis. This quantitative analysis serves to stratify the variants, distinguishing between those that are relatively common in clinical reporting and those that remain exceptionally rare.

## 3. Results

We identified 11 clinical presentations corresponding to rare and atypical variants of psoriasis (Table 1):

1. Psoriasis Gyrata (PG)
2. Linear Psoriasis (LP)
3. Follicular Psoriasis (FP)
4. Psoriatic Onycho-Pachydermo-Periostitis (POPP)
5. Psoriatic Neurodermatitis (PN)
6. Interdigital Psoriasis (IP)
7. Parakeratosis Pustulosa (PP)
8. Psoriasis of the lips
9. Psoriasiform acral dermatitis (PAD)
10. Psoriasis Dermatitis (PD)
11. Extreme hyperkeratotic forms

LP, FP, and PP emerged with the highest publication volumes. PG and PN, together with the extreme hyperkeratotic forms, demonstrated the lowest literature volumes.

### 3.1. Psoriasis Gyrata

**Figure 1:**
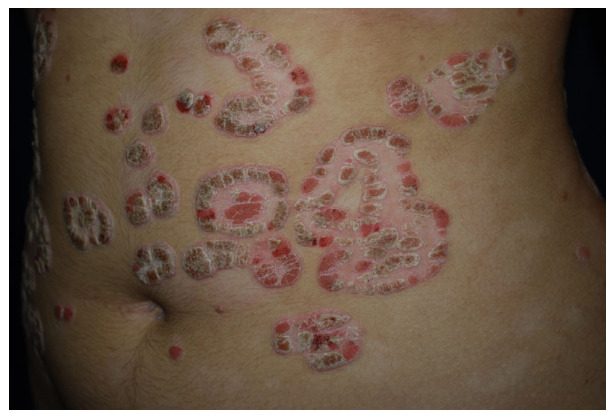
Clinical presentation of psoriasis gyrata characterized by polycyclic and serpiginous erythematous-desquamative plaques.

PG is a very particular form distinguished by the singular form and distribution of the scaly patches that constitute large arabesque-like lesions. These are tortuous and vermiform, or circular and semicircular with tortuous appendages, mainly affecting the trunk (Figure 1). These can be interpreted as the result of annular lesions evolving into polycyclic patterns [3].

The molecular mechanisms driving the concentric, gyrate expansion of lesions remain partially elusive. However, comparative studies on migratory patterns of T-cells and keratinocytes suggest a “reaction-diffusion” wave phenomenon. In PG, the local concentration of specific chemokines (such as CXCL8 and CXCL10) drives a rapid, centrifugal migration of neutrophils and Th17/Th1 cells. This outpaces the central resolution capacity of regulatory T-cells, leaving a cleared or less inflamed center and a highly active, advancing gyrate border [3].

The main differential diagnoses of PG include conditions with annular or polycyclic lesions such as tinea corporis, lichen planus annulare, nummular eczema, erythema annulare centrifugum, secondary syphilis, erythema marginatum, erythema gyratum repens (EGR), and erythema multiforme annulare. EGR is the most critical pitfall; unlike EGR, PG scales are typically micaceous and silvery, histological features remain strictly psoriatic, and its progression rate is significantly slower.

First-line therapies combine topical corticosteroids with vitamin D analogs and phototherapy; moderate-to-severe cases require systemic treatments, including biologics.

### 3.2. Linear Psoriasis

**Figure 2:**
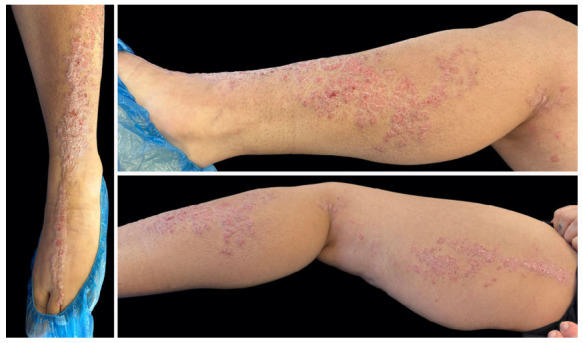
Linear psoriasis distributed along Blaschko’s lines.

LP is an exceptionally rare clinical variant of psoriasis, characterized by psoriatic plaques distributed along Blaschko’s lines, typically in a linear pattern (Figure 2). The condition exhibits two distinct profiles: isolated LP, not associated with classical psoriasis, and superimposed LP, occurring concomitantly with classical psoriasis [4]. Pustular LP, a rarer subtype, has also been observed [5]. The pathogenesis of LP remains unclear but is thought to involve genetic mosaicism. Happle’s theory suggests that linear manifestations result from a postzygotic mutation during embryogenesis, leading to loss of heterozygosity at a predisposing gene locus [4]. Genomic sequencing studies on microdissected linear tissue have confirmed this hypothesis. In patients with superimposed LP, next-generation sequencing has identified postzygotic somatic mutations in genes that regulate the IL-17/IL-23 axis or the NF-kB pathway (such as CARD14 or TRAF3IP2). In isolated LP, the mutation is confined strictly to the Blaschko-linear keratinocytes. In superimposed LP, a second-hit somatic mutation occurs on a background of an inherited, germline heterozygous predisposing mutation, sparking an exacerbated, treatment-resistant localized flare [6].

Distinguishing LP from other dermatoses in the Blaschko linear group is challenging. The main differential diagnosis is inflammatory linear verrucous epidermal nevus (ILVEN). Unlike LP, ILVEN typically presents at birth or during infancy, progresses slowly, and exhibits unilateral warty, pruritic lesions arranged in a linear pattern, often on the lower limbs. ILVEN lesions are more resistant to treatment compared to LP, which usually develops later in life, progresses rapidly, and responds to antipsoriatic therapy [4]. Other conditions, such as Blaschko-linear lichen planus or lichen striatus, can be ruled out with histological examination [3].

Local treatments, including topical corticosteroids and other antipsoriatic agents, are less effective in LP compared to classical psoriasis [7]. Also, biotechnological therapy has shown variable efficacy: linear lesions remain highly resistant, likely due to the altered, autonomous hyper-responsiveness of the mutated mosaic keratinocyte clone [4].

### 3.3. Follicular Psoriasis

**Figure 3:**
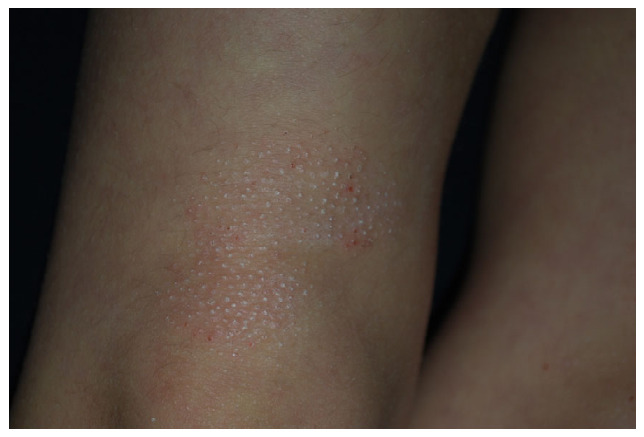
Follicular psoriasis presenting with clustered hyperkeratotic follicular papules.

FP is a rare and underreported clinical variant of psoriasis, originally described by MacLeod in 1921. It is characterized by hyperkeratotic follicular papules (Figure 3). This condition has been proposed as a potential initial form of psoriasis that may later progress to other subtypes or remain localized to follicular lesions for an extended period [8,9]. The pathogenesis of FP is not fully understood: it is thought to represent an early manifestation of psoriasis, with lesions originating around hair follicles.

Two clinical subtypes of FP have been described: the adult variant, predominantly affecting women, with discrete, follicular, hyperkeratotic papules concentrated on the thighs; and the variant affecting the trunk, axillae, and extensor aspects of the extremities, presenting as asymmetric, clustered follicular keratotic papules [10]. It is also described as flexural and mucosal involvement. Follicular lesions are sometimes seen adjacent to chronic plaque psoriasis, highlighting their potential relationship to more classic psoriatic presentations [11]. This condition is more frequently reported in children, although many adult cases are described [12].

Dermoscopy plays an essential role in identifying psoriatic follicular lesions. Typical dermoscopic findings in FP include a white-brown or pink-white perifollicular background, perifollicular scaling, and vascular features such as dotted vessels, twisted red loops, glomerular vessels, and red globules [10,11]. Histopathological findings include dilated follicular openings, follicular hyperkeratosis, perifollicular parakeratosis, parakeratotic plugging, hypogranulosis, Munro’s microabscesses, suprapapillary thinning, and tortuous, dilated upper dermal blood vessels [10,11,13]. The differential diagnosis of FP includes pityriasis rubra pilaris, lichen spinulosus, follicular eczema, keratosis pilaris, and follicular lichen planus [10].

Therapy begins with topical agents like steroid-salicylic acid combinations; for persistent or widespread cases, therapeutic approaches escalate to phototherapy and systemic drugs, including biologics [12].

### 3.4. Psoriatic Onycho-Pachydermo-Periostitis

**Figure 4:**
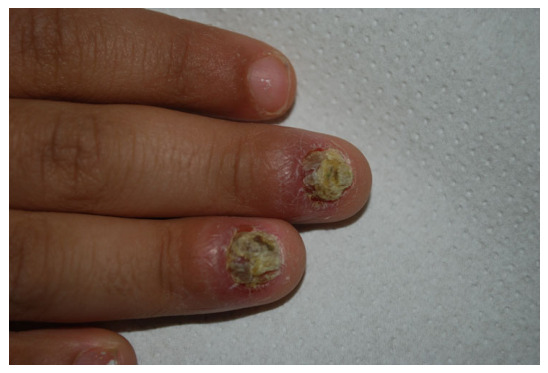
Psoriatic onycho-pachydermo-periostitis involving the distal phalanx and nail apparatus.

POPP, first described by Fournier in 1980, is a rare variant of psoriatic disease characterized by the isolated involvement of distal phalanges, particularly of the big toe [14]. Clinically, it presents with onychodystrophy, including onycholysis and longitudinal striations of the nail plate, painful swelling of the surrounding soft tissues (Figure 4), and acral bone changes [15]. Affected nails may exhibit oil spots and pitting [15].

In POPP, mechanical stress or micro-trauma triggers an innate immune response at the enthesis. The pathogenesis involves the extension of psoriatic inflammation from the nail bed to the underlying bone through entheses, resulting in periosteal thickening and osteitis similar to other psoriatic enthesopathies [16].

Scintigraphy is particularly sensitive in detecting early-stage disease, revealing intense focal uptake in the distal phalanx even when clinical and radiological findings are minimal. Radiological findings include solid periosteal reactions (and “tuft-like” or “brush-like” periosteal reactions) and acral bone thickening, often described as “ivory phalanx”. There is a total absence of involvement of the distal interphalangeal joints, distinguishing POPP from psoriatic arthritis [15,16]. An association with the HLA-B27 antigen and, more specifically, HLA-B39 has been noted, suggesting a genetic predisposition [16].

The differential diagnosis for POPP includes distal interphalangeal psoriatic arthritis, suppurative acrodermatitis continua of Hallopeau, gout, hypertrophic osteoarthropathy, and severe onychomycosis [3,15]. Therapeutic options for POPP include intralesional steroid injections into the nail matrix and systemic treatments like methotrexate and cyclosporine. In severe and recalcitrant cases, biological drugs (such as adalimumab, etanercept, secukinumab, or ixekizumab) show superior efficacy in halting both soft-tissue swelling and underlying bone remodeling [17].

### 3.5. Psoriatic Neurodermatitis

**Figure 5:**
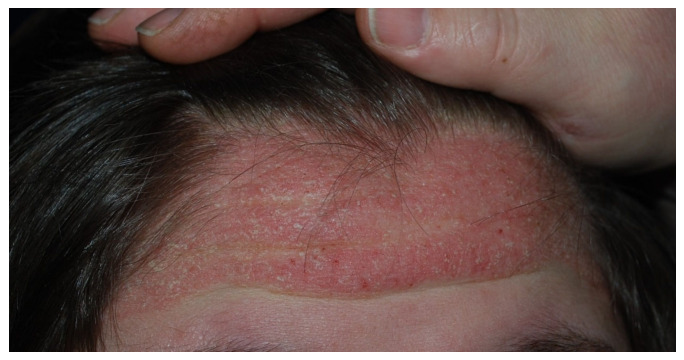
Psoriatic neurodermatitis with lichenified psoriatic plaques.

PN typically exhibits erythematous, scaly plaques localized predominantly on the extremities [18]. Unlike plaque psoriasis, PN plaques are fewer in number and smaller in size (median 4 cm). The hallmark feature of PN plaques is lichenification, which manifests as exaggerated skin lines forming a crisscross pattern (Figure 5). This lichenified texture is more typical of Lichen Simplex Chronicus (LSC) but is seen in PN without the associated excoriations that are commonly observed in LSC plaques.

A key distinction of PN is its anatomical distribution. Lesions are predominantly confined to the extremities, with bilateral involvement of the knees and elbows being the most frequent pattern. This contrasts with LSC, which often involves the nape of the neck, vulva, feet, and occasionally the anogenital region. Itching is a dominant symptom in both conditions, but patients with PN reported less severe pruritus during rest compared to those with typical LSC.

The pathogenesis of PN remains under debate, highlighting the complex interplay between the peripheral nervous system and immune-mediated cutaneous inflammation (the neuro-cutaneous axis). PN lesions reveal a significant upregulation of nerve growth factor and an increased density of calcitonin gene-related peptide-positive and Substance P-positive unmyelinated C-fibers.

PN may represent a form of LSC superimposed on plaque psoriasis or arise through the Koebner phenomenon in predisposed individuals with psoriasis, since dermoscopy of PN reveals features indicative of both LSC and psoriasis. These include scales, regular linear vessels, and hyperkeratosis. Histologically, PN plaques show several findings typical of psoriasis, including hyperkeratosis, confluent parakeratosis, hypogranulosis, acanthosis, and thinning of suprapapillary layers. PN plaques lacked hallmark features such as spongiform pustules and vertical thick collagen bundles typically observed in LSC.

Psychiatric comorbidities are notable in PN patients, above all depressive disorders and generalized anxiety [18].

### 3.6. Interdigital Psoriasis

**Figure 6:**
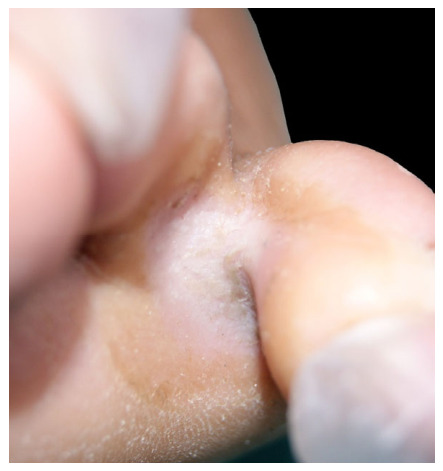
Interdigital psoriasis (“psoriasis alba”) involving interdigital toe spaces.

IP, first named ‘psoriasis alba’ by Waisman, represents a localized intertriginous form of psoriasis (commonly classified as psoriasis inversa). IP typically presents as whitish, sodden, sharply demarcated plaques or patches located between the toes (Figure 6) [19]. These lesions are firm, pliant, and leathery on palpation, distinguishing them from the soft, macerated appearance of interdigital fungal infection; however, mycological investigations, including direct microscopy and fungal cultures, are essential in ruling out infections [20]. Pruritus is either absent or mild, and patients often remain asymptomatic. The condition predominantly affects the intertoe spaces of one or both feet; remarkably, interdigital psoriasis is rarely observed between the fingers [20].

The unique localization to interdigital spaces may be influenced by friction, moisture, and maceration, potentially triggering the Koebner phenomenon [21]. Recent studies have revealed a complex bi-directional relationship: frictional trauma induces the psoriatic plaque with an altered, hyperkeratotic skin barrier; the compromised interdigital barrier allows for secondary fungal colonization, triggering an upregulation of IL-23 [22]. The persistent hydration alters the optical properties of the stratum corneum, causing the classic “whitish” and sodden appearance, which masks the underlying erythema typical of classic plaque psoriasis.

Histological examination of IP reveals features consistent with psoriasis, although with some variations. Common findings include confluent hyperparakeratosis, intermittent stratum granulosum, and psoriasiform hyperplasia. Occasionally, subcorneal micropustules (e.g., Kogoj pustules) may be observed, while Munro microabscesses are typically absent. Periodic acid-Schiff staining is negative, aiding in the exclusion of fungal infections [20].

The management of IP is similar to other forms of intertriginous psoriasis. Topical therapies, including corticosteroids and vitamin D analogs, are effective in most cases [22].

### 3.7. Parakeratosis Pustulosa

**Figure 7:**
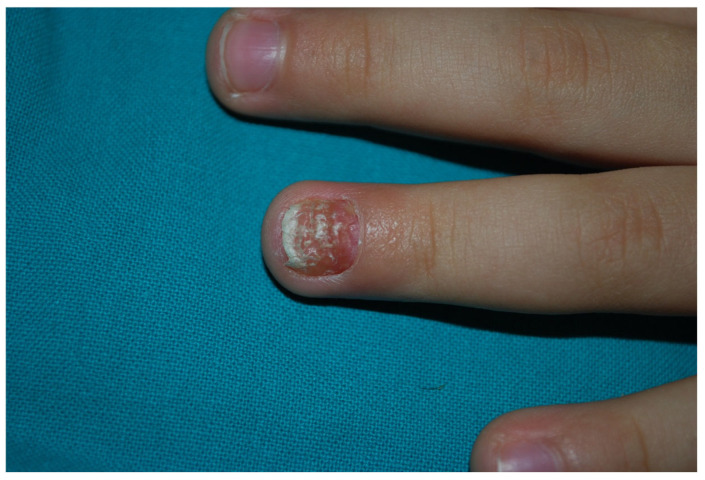
Parakeratosis pustulosa affecting the periungual region.

PP is a unique pediatric nail condition. The disease commonly affects a single finger, typically the thumb or index finger, and is characterized by mild eczematous changes in the fingertip pulp combined with psoriasiform alterations of the nail (Figure 7). The condition usually begins with pustules or vesicles on the pulp, progressing to mild eczematous changes, and occasionally leading to pulpitis. Nail changes, which typically follow the skin inflammatory phase, include mild subungual hyperkeratosis and onycholysis, often more pronounced on one side of the nail. Spontaneous remission is frequent [23]. Acrodermatitis continua of Hallopeau remains the most common misdiagnosis in these patients: it is more common in adults, though it can also be observed in childhood; it usually has a more aggressive course with the presence of pustular lesions, pain, potential nail deformity, and osteolysis; it rarely resolves spontaneously. The differential diagnosis for PP also includes onychomycosis, distinguished by fungal culture and microscopy; chronic paronychia, identified by persistent inflammation of the proximal nail fold, nail sucking, typically observed in younger children with evident behavioral habits, and eczema, which may overlap with PP, but patch testing can help rule out allergic contact dermatitis [23].

The progression of PP to nail psoriasis has been documented in some patients, suggesting a potential overlap between PP and early psoriatic nail disease [24].

### 3.8. Psoriasis of the Lips

**Figure 8:**
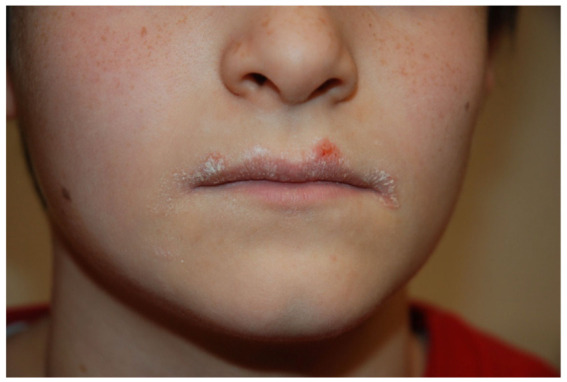
Psoriasis of the lips.

Psoriasis of the lips typically presents with scaling, fissuring, and erythema, often accompanied by discomfort such as a burning sensation and difficulty during eating or speaking. Lesions may involve one or both lips, with extension over the vermilion border (Figure 8), while the remaining oral mucosa generally appears unaffected. In some cases, lip involvement may occur in association with other psoriatic lesions [25].

Dermoscopy of the lip lesions demonstrates diffuse, monomorphic dots and globular vessels, accompanied by white scales [25]. Psoriasis of the lips, however, can be clinically misdiagnosed as chronic eczema, actinic cheilitis, chronic candidiasis, or leukoplakia. The white color of psoriatic scales in dermoscopy, in contrast to the yellow scales of dermatitis, is a particularly valuable diagnostic clue [26].

Since 2000, only fifteen cases of psoriasis affecting the lips have been identified through our screening methodology. Psoriasis of the lips can precede or follow the appearance of classic psoriatic lesions.

### 3.9. Psoriasiform Acral Dermatitis

The term PAD was coined by Zaias in 1980 [27,28,29]. PAD is a rare and distinctive form of dermatitis that primarily affects the distal phalanges of the fingers, with a notable shortening of the nail bed. The lesions are typically psoriasiform in nature, characterized by erythema, scaling, and fissures on the palmar surfaces of the digits, often accompanied by a sclerodermoid appearance of the dorsal aspects of the fingers [27].

While PAD may present isolated, it can also occur in conjunction with typical psoriatic lesions in other areas of the body. It has been primarily observed in children. The differential diagnosis of PAD includes other acral dermatitis conditions such as contact dermatitis, dyshidrotic eczema, and atopic dermatitis. However, the absence of a personal or family history of atopy, negative patch tests, and normal serum IgE levels in the observed cases point against these diagnoses [28].

Histologically, PAD presents as a subacute dermatitis with a dense lymphocytic infiltrate, massive spongiosis, parakeratosis, scale crusts, psoriasiform acanthosis, and exocytosis; this has led some to consider PAD a separate entity, distinct from psoriasis [28]. However, the concurrent presence of classic psoriatic lesions elsewhere in the body, along with nail dystrophy, suggests that PAD may in fact be a particular variant of acral psoriasis in children [27].

The pathogenesis of PAD is not fully understood; it may be genetically predisposed, as it frequently occurs in children who later develop typical psoriasis lesions in other areas of the body. Environmental factors, such as seasonal changes, seem to influence the severity of PAD, with some patients showing improvement during summer holidays at the seaside [27]. The pathogenesis of nail shortening observed in PAD is believed to be related to hypertrophy of the cuticle, secondary to the inflammation, rather than a permanent anatomical change in the nail bed [27].

While it is often resistant to treatment and can cause significant disability when multiple fingers are involved, it may resolve spontaneously during puberty [27].

### 3.10. Psoriasis Dermatitis

PD is a condition characterized by a blend of clinical features of both psoriasis and atopic dermatitis (AD). The condition is particularly observed in children, and its understanding is evolving as researchers explore the overlap between these two diseases. It has been proposed as a spectrum of disease rather than a distinct pathology, possibly due to shared immune activation mechanisms between AD and psoriasis. PD represents an intermediate phenotype between these two disorders, manifesting features from both [30,31,32,33].

Children with PD often present with facial lesions, a feature more commonly associated with AD, while the scalp is less frequently affected compared to children with psoriasis. Lesions may also occur on the body, resembling psoriasis, but without the classic well-demarcated plaques. The presence of eczema-like changes in the acute phase of PD, along with psoriasiform changes later, is a key feature distinguishing it from both pure AD and psoriasis [32]. Additionally, lesions can be found on areas that typically present with psoriasis, such as elbows, knees, and the torso [30,31].

Although psoriasis involves type 17 immune responses, atopic dermatitis is centered on type 2 and type 22 responses, with common type 1 polarization in chronic lesions. Recently, it has been observed that some cytokines involved in the pathogenic mechanisms of psoriasis (IL-17, IL-17C, and IL-23) are also implicated in the chronic phase of atopic dermatitis, as evidenced by the effectiveness of some psoriasis-targeted drugs, such as ustekinumab, in the treatment of moderate to severe chronic AD [34,35]. While Th17 cells drive inflammation in psoriasis and Th2 cells are central to AD, evidence suggests a concurrent activation of Th17 and Th2 pathways in PD. This mixed immune response could explain the overlapping features, where skin barrier dysfunction typical of AD is coupled with inflammatory processes seen in psoriasis [32,33,34,35].

Unlike psoriasis, nail involvement appears to be absent in PD. Conversely, involvement of the feet, glutes, and genitalia is more common than in AD, while facial and neck lesions are less frequent [34]. Histological findings in PD are limited but suggest that PD shares features of both psoriasis and AD. Skin biopsies often demonstrate psoriasiform hyperplasia, hypogranulosis, spongiosis, and eosinophilic infiltrates [34]. Moreover, studies suggest that metabolic comorbidities, such as obesity, are more common in PD patients compared to those with either psoriasis or AD alone.

The diagnosis of PD remains challenging due to its mixed features and lack of universal criteria, often relying on clinical features. In the differential diagnosis of psoriasiform dermatitis (PD), the findings most suggestive of psoriasis include erythro-squamous plaques on the body and/or extremities, pustules excluding facial pustules, and erythema of the anal cleft. Conversely, features more supportive of atopic dermatitis include eczema and/or lichenification of the flexural areas, dyshidrotic eczema, and Dennie–Morgan folds and/or periorbital darkening. Table 2 reports the complete ranking of clinical features suggestive of psoriasis and atopic dermatitis, according to Müller et al. [35,36].

With regard to treatment, although randomized controlled trials are still needed to define the optimal therapeutic approach for PD, a practical decision-making algorithm has recently been proposed. This algorithm takes into account different clinical scenarios according to whether PD is primary or induced by biologic therapies targeting the Th1/Th17 or Th2 pathways [36].

Extreme hyperkeratotic variants of psoriasis are characterized by a marked accumulation of thick, adherent scales and are often relatively resistant to conventional topical therapies. In the literature, the terms used to describe these forms are sometimes applied interchangeably; however, several clinical features may help distinguish rupioid, ostraceous, and elephantine psoriasis.

Rupioid psoriasis derives its name from the Greek word *rhupos*, meaning filth or dirt, and was first described in 1948. Clinically, it is characterized by dark, extremely thick, well-demarcated hyperkeratotic plaques arranged in concentric layers. These lesions often assume a conical morphology, resembling a limpet shell. The “dirty” appearance reflects the presence of thick crusts, serosanguineous exudate, and marked epidermal hyperplasia. Recent observations suggest that psoriasis with very thick plaques may show a male predominance and may be associated with a higher frequency of psoriatic arthritis and nail involvement, as well as a greater affected body surface area compared with thin-plaque psoriasis [37,38].

Ostraceous psoriasis shares several features with rupioid psoriasis but typically presents with oval, circular, or ring-like hyperkeratotic plaques with a concave surface, closely resembling an oyster shell. The scales are usually thick, firmly adherent, and difficult to remove. This variant is also considered poorly responsive to topical treatment and has been reported in association with psoriatic arthritis [39].

**Figure 9:**
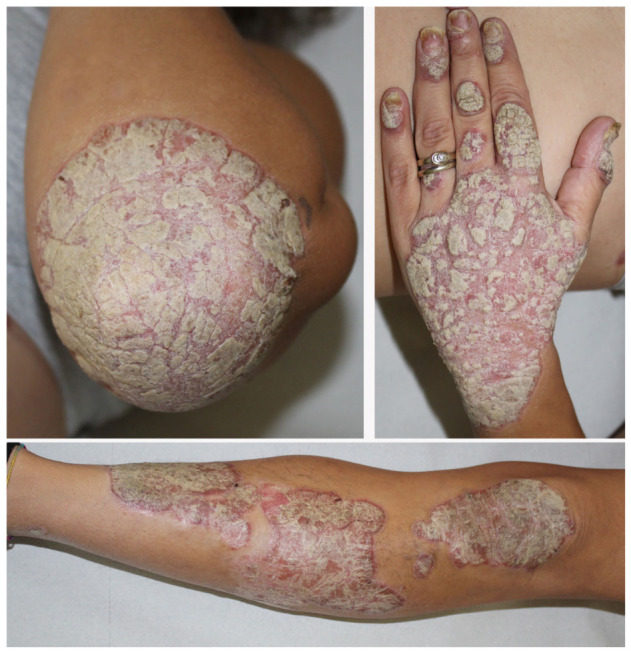
Elephantine psoriasis.

Elephantine psoriasis represents another severe hyperkeratotic phenotype, presenting with exceptionally thick, extensive, silvery-white scaly plaques that may cover large areas of the body and resemble elephant skin (Figure 9). Lesions are most frequently described on the back, buttocks, upper limbs, and lower limbs. Histopathological findings may include marked papillomatosis and alternating areas of hypogranulosis and hypergranulosis [40].

A key differential diagnosis of hyperkeratotic psoriasis is ILVEN; although ILVEN typically follows the lines of Blaschko, it shares many histological features with psoriasis, specifically the presence of alternating bands of hypergranulosis and hypogranulosis. For lesions displaying a rupioid morphology, clinicians must also consider secondary or late latent rupioid syphilis, keratotic scabies, reactive arthritis, and disseminated histoplasmosis [41]. Other reported differential diagnoses for these hyperkeratotic forms include verrucous Darier’s disease, acquired palmoplantar keratoderma, and photosensitive skin lesions associated with aminoaciduria.

## 4. Conclusions

Rare and atypical variants of psoriasis remain diagnostically challenging because of their unusual morphology, atypical localization, and overlap with numerous inflammatory and infectious dermatoses. Although an experienced dermatologist should be able to recognize most presentations, some variants may require additional investigations to avoid diagnostic pitfalls. Dermoscopy, reflectance confocal microscopy, and optical coherence tomography can provide valuable diagnostic support; nevertheless, histopathological examination remains the gold standard when clinical findings are inconclusive.

Accurate recognition of these uncommon forms is clinically important, as misdiagnosis may lead to unnecessary investigations and inappropriate treatments, particularly repeated courses of antifungals or antibiotics. Moreover, certain variants, such as psoriatic onycho-pachydermo-periostitis, may signal underlying musculoskeletal involvement requiring multidisciplinary management.

The pathogenesis of these rare presentations remains incompletely understood and may involve mechanisms such as genetic mosaicism, Koebnerisation, follicular-centered inflammation, or localized enthesopathy. Whether they represent distinct pathogenetic entities or simply phenotypic expressions within the psoriatic spectrum remains to be clarified.

Dermatology is a profoundly visual specialty, confirming the well-known axiom that “you diagnose what you already know” [42]. The peculiar clinical features reviewed here support this concept: once recognized, these variants become considerably easier to identify. However, current therapeutic evidence remains limited, being derived largely from case reports and small case series, highlighting the need for multicenter studies to establish evidence-based diagnostic and treatment strategies.

## Figures and Tables

**Table 1:** Main features of atypical and rare forms of psoriasis.

	Clinic	Dermoscopy	Location	Differential Diagnosis	Histology	Pathogenesis	Diagnosis
PG	Scaly tortuous, vermiform, circular, or semicircular patches with tortuous appendages	Not described, probably common psoriasis-like	Mainly the trunk	tinea corporis, lichen planus annulare, nummular eczema, contact dermatitis, erythema annulare centrifugum, pityriasis rosea, secondary syphilis, parapsoriasis, cutaneous lupus erythematosus, erythema multiforme annulare	Not described, probably common psoriasis-like	The result of annular lesions evolving into polycyclic patterns	clinic
LP	Lesions resemble classical psoriasis, presenting as erythematosquamous plaques. Pruritus is uncommon. A pustular variant of LP is even rarer.	Not described, probably classical psoriasis-like	Along Blaschko’s lines, typically in a linear pattern	inflammatory linear epidermal nevi, Blaschko-linear lichen planus, or lichen striatus	Common psoriasis-like	The pathogenesis of LP is thought to involve genetic mosaicism, where postzygotic mutations during embryogenesis lead to a segmental manifestation of psoriasis. Loss of Heterozygosity (LOH): Postzygotic mutations may result in LOH at one or more psoriasis-predisposing gene loci, contributing to the linear distribution of lesions	Clinic histology
PF	hyperkeratotic clustered papules alone or alongside plaque-type psoriasis	white-brown or pink-white perifollicular background, perifollicular scaling, and vascular features such as dotted vessels, twisted red loops, glomerular vessels, and red globules	Thighs, trunk, axillae, and extensor limbs. Also described flexural and mucosal involvement	Follicular lichen planus and pityriasis rubra pilaris: Guttate psoriasis, papular pityriasis rosea, papular secondary syphilis, pityriasis lichenoides chronica	Follicular involvement: dilated follicular openings, follicular hyperkeratosis, perifollicular parakeratosis. Parakeratotic plugging, hypogranulosis, Munro’s microabscesses, suprapapillary thinning, and tortuous, dilated upper dermal blood vessels	Not fully understood; early manifestation of psoriasis, with lesions originating around hair follicles	clinical, supported by dermoscopy and histopathological findings
POPP	Onycholysis and longitudinal striations of the nail plate. Painful swelling of the surrounding soft tissues. Erythema, non-scaly in most cases.	Not explicitly detailed, but the nail findings include: oil spots, pitting, and nail dystrophy.	distal phalanges, predominantly in the great toes (often bilateral)	Psoriatic arthritis involving distal interphalangeal joints. Hallopeau’s acrodermatitis continua. Onychomycosis Gout Primary hypertrophic osteoarthropathy.	Not described	Localized Enthesopathy: inflammation spreads from the psoriatic nail to adjacent bone, causing periosteal reactions and hyperostosis. Genetic Links: associated with HLA-B27 and HLA-B39, indicating genetic predisposition.	Based on clinical, radiological, Radiography shows classic “ivory phalanx” (periosteal thickening). Brush-like or tufted appearances at the distal phalanx. Early-stage involvement can be detected via bone scintigraphy
PN	keratotic lichenified plaques with sharp borders but less excoriated than LSC	No specific dermoscopic features mentioned	on the extremities, particularly the knees and elbows, Rarely on the head and neck.	Plaque Psoriasis Lichen Simplex Chronicus	Hyperkeratosis, Confluent Parakeratosis, Hypogranulosis: Microabscesses in the Horny Layer: Acanthosis, Thinning of Suprapapillary Plates: PN plaques lacked hallmark features such as spongiform pustules and vertical thick collagen bundles typically observed in LSC	Two hypotheses for PN pathogenesis: (1) Development of psoriasis due to the Koebner phenomenon from prolonged rubbing in a patient with LSC. (2) Superimposition of LSC on itchy psoriatic lesions	Clinical and histopathological findings. Family history of psoriasis supports the diagnosis of PN
IP (PA)	whitish, sodden plaques or patches, sharply demarcated. Not itchy, and the skin appears dry and pliable to the touch	Not described	interdigital spaces of the feet. but can extend to the plantar surface in some cases.	interdigital fungal infections (IFI), eczema, other forms of psoriasis, and contact dermatitis.	acanthosis, granular layer alterations, parakeratosis, Kogoj micropustules, perivascular lymphocytic infiltrate, absence of Munro abscesses. Negative PAS staining.	The Koebner phenomenon, where psoriasis lesions appear in areas of skin trauma, may also play a role, particularly if there is pre-existing fungal infection.	clinical examination, histological analysis, and exclusion of other conditions. (fungal cultures and swab tests)
pp	pustules or vesicles progressing to mild eczematous changes, and occasionally leading to pulpitis. Nail changes, include mild hyperkeratosis and onycholysis	Not described	Fingertip pulp, nails and periungual areas, often localized to a single finger, typically the thumb or index finger.	Onychomycosis Nail sucking Chronic paronychia Eczema	Not described	It may develop due to inflammation in the periungual area or be associated with other forms of dermatitis.	clinical features and the exclusion of other conditions.
Psoriasis of the lips	caling and cracking of the lips, accompanied by a burning sensation and severe discomfort while eating and speaking	dots and globular vessels that were diffuse and monomorphic, accompanied by white scales	lips were involved, with lesions affecting both the upper and lower lips and extending over the vermilion border	Chronic eczema Actinic cheilitis Chronic candidiasis Leukoplakia	not described	specific pathogenesis for lip involvement is not detailed in the text.	clinical presentation and dermoscopic findings.
PAD	erythema, scales, and fissures on the distal phalanges of the fingers. The nail bed was shortened and tapered, and the cuticle extended onto the nail plate in all affected digits. Some patients exhibited a sclerodermoid appearance on the dorsal surface of involved fingers	not specifically described	distal phalanges of the fingers, with some involvement of the dorsal surface of the fingers. The palmar surface was involved in a minority of cases.	Contact dermatitis Atopic dermatitis Dyshidrotic eczema Psoriasis	Subacute dermatitis with a dense lymphocytic infiltrate Massive spongiosis Parakeratosis with scale crusts Psoriasiform acanthosis Exocytosis	The pathogenesis of PAD remains unclear, but the condition may represent a variant of acral psoriasis.	clinical features, histological findings, and the exclusion of other conditions.
PD	pruritus, erythematous patches, and eczema-like lesions with scaling plaques	not explicitly discussed in the provided articles; it can be inferred that dermoscopic evaluation in PD may show features from both psoriasis and AD	Face, scalp, trunk, limbs	AD Psoriasis Seborrheic Dermatitis Contact Dermatitis	In PD, histology shows psoriasis-like hyperkeratosis and spongiotic changes. Inflammatory infiltrates.	Psoriasis and AD involve immune dysregulation with different profiles: Th1/Th17 cells in psoriasis and Th2/Th22 cells in AD. Overlap syndrome (PD) suggests these conditions can coexist, with IL-17 playing a key role in PD pathogenesis.	Diagnosis is clinical and rarely histological

PG = psoriasis gyrate; LP = linear psoriasis; PF = follicular psoriasis; POPP = psoriatic onycho-pachydermo-periostitis; PN = psoriatic neurodermatitis, IP = interdigital psoriasis (psoriasi alba); pp = parakeratosis pustulosa; PAD = psoriasiform acral dermatitis; PD = psoriasis dermatitis; AD = atopic dermatitis.

**Table 2:** Ranking of Psoriasis and Atopic Dermatitis Clinical Features.

	Psoriasis	Atopic Dermatitis
1	Erythrosquamous plaques on the body and/or extremities *	Eczema and/or lichenification of the flexors *
2	Pustules (facial pustules excluded) *	Dyshidrotic eczema *
3	Erythema of the rima ani	Dennie–Morgan fold and/or orbital darkening
4	Scalp involvement beyond the forehead hairline	Perlèche (angular cheilitis) and/or cheilitis *
5	Plaque psoriasis localized retroauricular	Head and neck dermatitis and/or dirty neck
6	Psoriatic nail changes (pitting, oil-drop spots, nail plate crumbling)	Keratosis pilaris
7	Dactylitis/enthesopathy	Personal history for atopy (AST, AD, ARC)
8	Exacerbation after discontinuation of systemic steroid therapy	Sensitizations/food allergies
9	Family history of psoriasis	Palmar hyper-linearity
10	Joint pain	Family history of atopy (AST, AD, ARC, “eczema”)

* = currently or in the past; AD = atopic dermatitis; AST = asthma, ARC = allergic rhinoconjunctivitis.

## Data Availability

No new data were created or analyzed in this study. Data sharing is not applicable to this article.

## Abbreviations

The following abbreviations are used in this manuscript:

PG	Psoriasis gyrata
LP	Linear psoriasis
ILVEN	inflammatory linear verrucous epidermal nevus
FP	Follicular psoriasis
POPP	Psoriatic onycho-pachydermo-periostitis
PN	Psoriatic neurodermatitis
LSC	Lichen Simplex Chronicus
IP	Interdigital psoriasis
PP	Parakeratosis pustulosa
PAD	Psoriasiform acral dermatitis
PD	Psoriasis dermatitis
AD	Atopic dermatitis
